# Spontaneous Charge Generation in Flowing Albumin Solutions at 35 °C and 38 °C

**DOI:** 10.3390/bios7040060

**Published:** 2017-12-11

**Authors:** Yuri D. Ivanov, Andrey F. Kozlov, Rafael A. Galiullin, Ekaterina F. Kolesanova, Tatyana O. Pleshakova

**Affiliations:** Institute of Biomedical Chemistry, 119121 Moscow, Russia; afkozlow@mail.ru (A.F.K.); rafael.anvarovich@gmail.com (R.A.G.); ekaterina.kolesanova@ibmc.msk.ru (E.F.K.); t.pleshakova@gmail.com (T.O.P.)

**Keywords:** analytical flow-through systems, flowing protein solution, charge accumulation, serum albumin, physiological temperature

## Abstract

The time dependence of a charge accumulation in a 10^−15^ M albumin solution, flowing through a measuring cell of an analytical flow system injector, had a nonlinear character under certain conditions, within a human physiological temperature range. Sharp charge increases depended on albumin concentration. This effect must be taken into consideration when generating models that describe electrokinetic phenomena in flowing protein solutions and when developing analytical flow systems for the registration of biomolecules in low concentration ranges.

## 1. Introduction

The emergence of a charge generation effect that prevents correct measurements in nanowire biosensors is well known with respect to flow-through biosensors and to biosensors with protein solution injections via standard pipetting [1]. Attempts to eliminate these effects by including systems of charge withdrawal from a biosensor measurement unit have been made ever since the development of measurement schemes in these biosensors [1]. The charge generation effect in biosensor injector systems needs to be thoroughly studied, as this effect must be taken into account in scheme development and when electric measurements in nanowire biosensors are conducted. 

Besides that, charge generation has been observed in efficient protein fishing in atomic force microscopy (AFM) biosensors in extremely low (femtomolar and sub-femtomolar) concentrations [2,3]. It was noted that, in a biosensor system injector, low-copy protein (e.g., albumin in highly diluted solutions, as a model) registration efficiency dropped sharply when the charge generation velocity decreased [2]. There is an interesting publication describing the use of Coulometers, which measure hydrogel charge states, monitoring the binding of charged drug molecules to albumin in a protein hydrogel sensor [4]. Hence, charge generation can be observed in the majority of analytical systems, including diagnostic protein-based ones, in which the main unit is represented by an injection block for analyte delivery to a sensing element [2,3,5,6,7,8,9]. Thus, there is a need to study the nature of charge generation and the possibility of manipulating the charge value in the injection systems of biosensors. 

It is well known that a flowing liquid elicits electrokinetic phenomena at the interface, which are manifested in particular as an emergence of a difference in potentials. The binary electric layer that forms at the interface plays an important role in this event [10]. 

The mechanism of charge generation in liquid flow is generally associated with triboelectric effects, but it is not completely understood [11]. The discussion in [11] states that there may be either total or partial charge leak upon the solution flow into a measuring cell through a pipet tip, which can influence the efficiency of biosensor measurements, and this issue requires clarification. 

In the present study, we used the injector part of a flow-through AFM fishing system, described in [2,12], to monitor protein solution electrization in a measuring cell while the protein solution (i.e., a bovine serum albumin (BSA) solution) was pumped through the pipet tip. We have used this injector system earlier in AFM fishing measurements and for testing other analytic biosensor systems. A sharp charge increase, in addition to the commonly observed slow linear charging, was demonstrated in one of the same experiment. We observed no charge accumulation in the measuring cell at a higher albumin concentration (10^−4^ M), which was close to the protein concentration of human blood. Measurements were performed at 35 °C, correspondent to decreased human body temperatures, and at 38 °C, correspondent to elevated human body temperatures. We chose these temperatures, because erythrocyte fluidity characteristics are stable at 35 °C and at 38 °C, contrary to 36.6 °C, at which these characteristics are unstable [13]. 

## 2. Materials and Methods

Deionized water (resistivity 18 MΩ·cm) was obtained with the help of the Millipore Simplicity UV system (Molsheim, France).

Fatty acid-free BSA (Sigma, St. Louis, MO, USA, cat. No. A6003) was dissolved in deionized water at 10^−4^ M concentration. 10^−15^ M BSA solution was prepared by sequential dilutions. 

**Figure 1:**
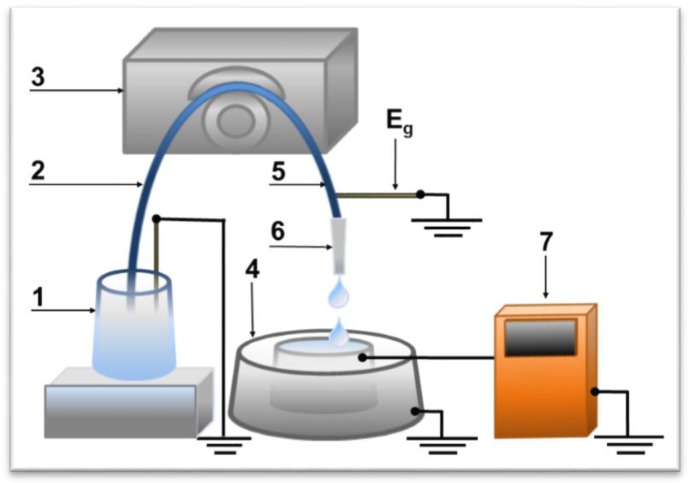
Block scheme of the charge measuring device in the solution, permanently delivered to the AFM-fishing system measuring cell: 1—a grounded container with the flowing solution; 2—silicone tubing (the input part); 3—peristaltic pump; 4—measuring cell linked to an electrometer; 5—silicone tubing (the output part); 6—a tip; 7—electrometer Keithley 617, Eg—grounding electrode.

Figure 1 shows the scheme of the charge measuring device. Electric charge measurements were performed with the help of an electrometer combined with the flow-through sample delivery system for AFM fishing [2,12] (Figure 1). 

Principal units of the sample delivery system included a peristaltic pump, a tubing with a tip for liquid delivery, and a measuring cell. Upon measurements, water or protein solution (10^−15^ M or 10^−4^ M) was permanently delivered with the help of the pump (Ismatech (IDEX)) (3) from a 50 mL polypropylene tube (1) with a grounding stem inside to the measuring cell (4). A sterile silicone tubing (length 40 cm, i.d. 2.0 mm) (5) with a standard disposable automatic pipet tip (for a 1–10 μL pipet, i.d. 0.4 mm) (6) was used for the water and protein solution delivery. Flow speed (~15 μL/s) was chosen so that drops (ca. 15 μL) were formed on the tip outlet. The grounding electrode Eg was put into the delivering tubing to support the solution potential at a constant level. The distance between the electrode and the tip outlet edge was 15 cm. Silicone tubing and the tip were exchanged for new ones after each experimental series, i.e., a set of parallel measurements made for the same protein solution or water at a certain temperature point. 

The stainless steel inner cylinder (4) represents a measuring cell in the charge measurement system, connected to the electrometer (7). Registration accuracy was 0.1 nC.

The measuring system was filled with water or protein solution from the tube (1) and washed with about 10 mL of this liquid before each experiment series. After that, the pump was switched off, the tip was directed to the measuring unit, the pump was switched on again simultaneously with the electrometer, and water or protein solution started to enter dropwise into the cell. At this moment, parallel experiment time counting and charge measurement data recording began. Records were made every 15 s for a period of 7 min. After each experimental series the measuring cell was drained and the electrometer was set to “0”.

The data are presented as the charge value over the baseline, ∆q, inside the measuring cell at the corresponding measurement time. Data curves are numbered in chronological order. 

## 3. Results

### 3.1. Monitoring Charge Generation and Accumulation in Water and Protein Solution at 35 °C

**Figure 2:**
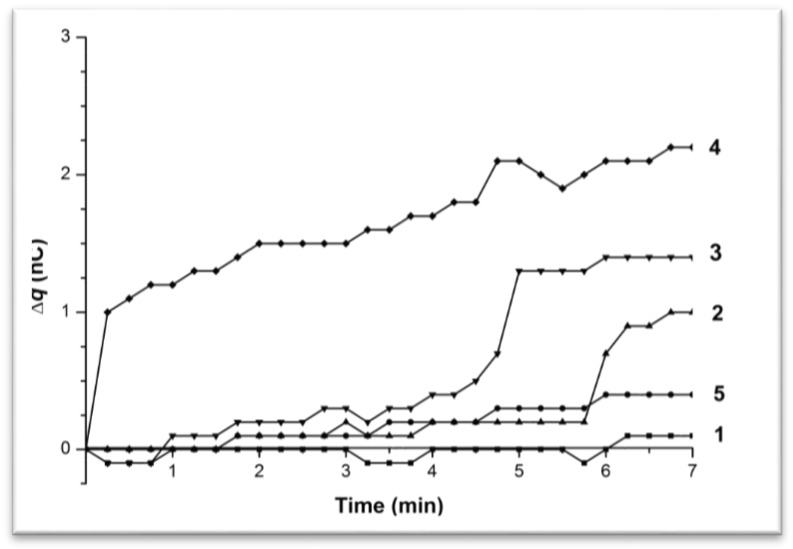
Time dependence curves of charge accumulation ∆q(t) upon water influx from the tip in the flow-through system at 35 °C. Curves 1–5 represent parallel measurements of one and the same experimental series.

Figure 2 shows time dependence curves of the charge generation and its accumulation in the cell (∆q) upon water pumping through the tip at 35 °C. 

One can observe the time-dependent increase of the charge value inside the measuring cell upon water drops into it. The maximal overall charge increase during the measurement time (7 min) was about 2 nC, that is, charge accumulation occurred. Charge value changes had either linear (Figure 2, Curve 5, linear increase in charge value with ∆q/∆t = 0.4/7 = 0.06 nC/min) or irregular (linear with a sharp increase—Figure 2, Curves 3–5) character. Curve 3 showed a smooth elevation of the charge during the initial 6 min (∆q/∆t ~ 0.2/6 min = 0.03 nC/min) followed by a sharp rise of 0.7 nC in a 30 s interval (∆q/∆t ~ 1.4 nC/min). Curve 4, similar to Curve 3, showed a smooth elevation of the charge value with ∆q/∆t 0.09 nC/min in the 0–4.25 min interval followed by the sharp rise of the charge for 0.8 nC in 30 s (∆q/∆t ~ 1.6 nC/min). Curve 5 also represented an example of the linear-sharp changing charge value. However, in this case, the sharp rise of 1 nC was observed during the first 15 s (∆q/∆t ~ 4 nC/min) followed by the smooth increase with the speed of 0.3 nC/min. Curve 1 represented a case with no noticeable charge changes. 

**Figure 3:**
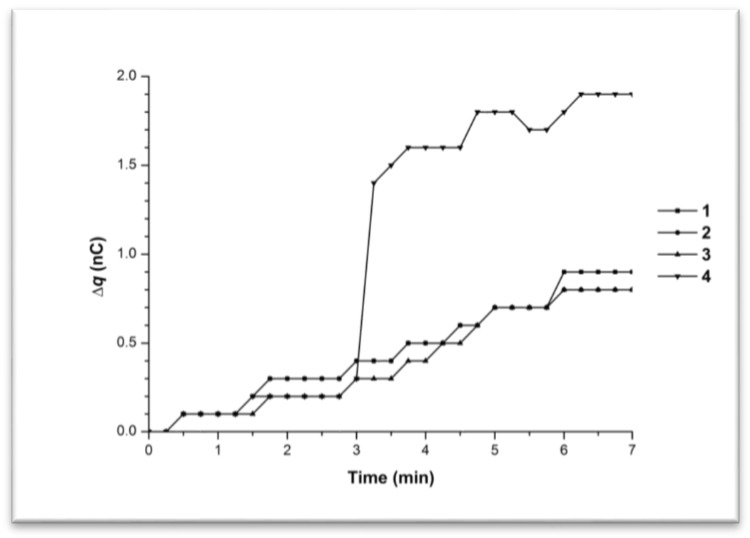
Time dependence curves of charge accumulation ∆q(t) upon 10^−15^ M BSA solution influx from the tip in the flow-through system at 35 °C. Curves 1–4 represent parallel measurements of one and the same experimental series.

Figure 3 shows time dependence curves of the charge generation and its accumulation in the cell (∆q) upon pumping the 10^−15^ M BSA solution at 35 °C.

One can see that the maximal overall charge increase during the measurement time (7 min) was about 2 nC, the same as for the water influx. Charge accumulation in the measuring cell had either a linear (Figure 3, Curves 1–3, ∆q/∆t ~ 0.1 nC/min) or a linear-to-abrupt (Figure 3, Curve 4) time dependence. As for Curve 4, an initial slow linear increase of the charge value with ∆q/∆t ~ 0.13 nC/min was observed for 3 min, followed by the sharp rise of 1.1 nC for 15 s (∆q/∆t ~ 4 nC/min) with a further smooth increase of 0.4 nC for 4 min (∆q/∆t ~ 0.1 nC/min). 

Hence both water and the BSA solution influx at 35 °C resulted in charge generation and accumulation in the measuring cell with a linear-to-abrupt time dependence of charge accumulation. 

**Figure 4:**
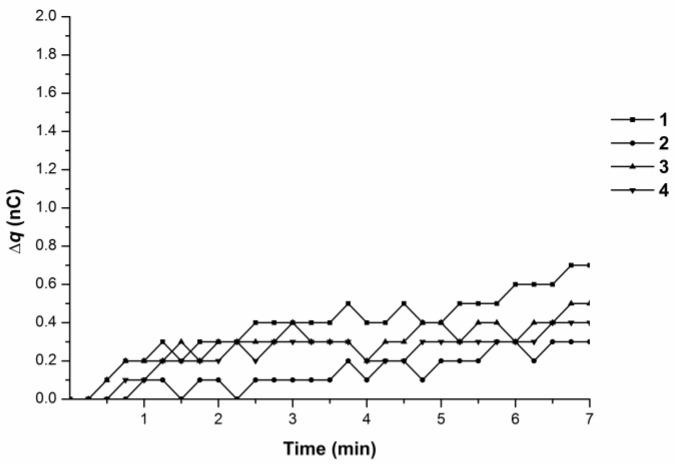
Time dependence curves of charge accumulation ∆q(t) upon 10^−4^ M BSA solution influx from the tip in the flow-through system at 35 °C. Curves 1–4 represent parallel measurements of one and the same experimental series.

Experiments with the 10^−4^ M BSA solution showed no abrupt charge accumulation in the measuring cell (Figure 4). 

### 3.2. Monitoring Charge Generation and Accumulation in Water and Protein Solution at 38 °C

**Figure 5:**
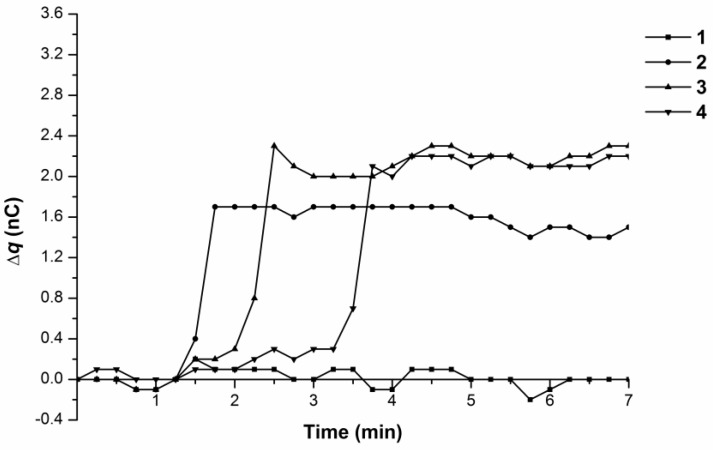
Time dependence curves of charge accumulation ∆q(t) upon water influx from the tip in the flow-through system at 38 °C. Curves 1–4 represent parallel measurements of one and the same experimental series.

Figure 5 shows the time dependence curves of the charge generation and its accumulation in the cell (∆q) upon water pumping through the tip at 38 °C.

Maximal overall charge value change was 2.8 nC according to the 7-min-interval observations, i.e., charge accumulation occurred at 38 °C and at 35 °C. We observed the linear-to-abrupt character of the time dependence of charge accumulation in the measuring cell. Curve 1 represented the case, when no charge drained into the cell upon liquid influx. The remaining curves showed an initial smooth increase in charge accumulation from 0 to 0.4 nC for 1.25–3.25 min (i.e., ∆q/∆t ~ 0.12 nC/min), followed by a sharp change of 3 to 4 nC/min. 

Maximal overall charge value change was 2.8 nC according to the 7-min-interval observations, i.e., charge accumulation occurred at 38 °C and at 35 °C and was similar. We observed a similar linear-to-abrupt time dependence of charge accumulation in the measuring cell. Curve 1 represents the case when no charge drained into the cell upon liquid influx. The remaining curves showed an initial smooth increase in charge accumulation from 0 to 0.4 nC for 1.25–3.25 min (i.e., ∆q/∆t ~ 0.12 nC/min), followed by a sharp increase of 3–4 nC/min. 

**Figure 6:**
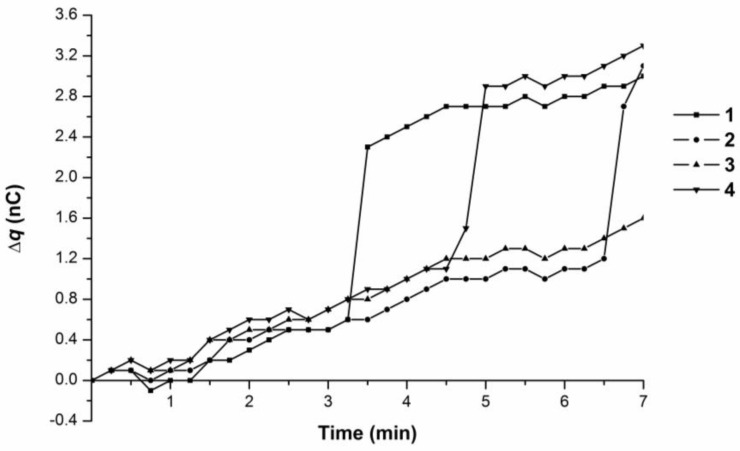
Time dependence curves of charge accumulation ∆q(t) upon 10^−15^ M BSA solution influx from the tip in the flow-through system at 38 °C. Curves 1–4 represent parallel measurements of the same experimental series.

Figure 6 contains the time dependence curves of the charge generation and its accumulation in the measuring cell as the 10^−15^ M BSA solution was pumped at 38 °C. 

Maximal overall charge value change was 3.5 nC according to the 7-min-interval observations, which again indicated charge accumulation. The observed charge value change corresponded to either linear (Curve 3 with ∆q/∆t ~ 0.2 nC/min) or linear-to-abrupt (Curves 1, 2, and 4) time dependences. Curves 1, 2, and 4 were characterized by an initial smooth charge accumulation from 0 to 1.1 nC for 3–6.5 min (i.e., ∆q/∆t ~ 0.2 nC/min), followed by a sharp increase of 1.8–2.0 nC for 15 s (i.e., ∆q/∆t from 7.2 to 8.0 nC/min). 

Hence, both water and the 10^−15^ M BSA solution, at 38 °C and at 35 °C, were characterized by the generation of a charge during their influx into the measuring cell from the plastic micropipet tip. The time-dependent accumulation of this charge demonstrated either a linear or a linear-to abrupt character at both temperatures, 35 °C and 38 °C. 

**Figure 7:**
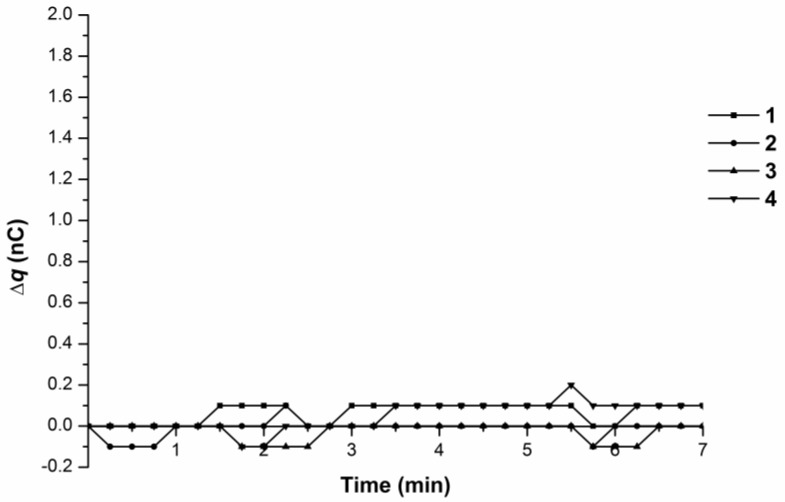
Time dependence curves of charge accumulation ∆q(t) upon 10^−4^ M BSA solution influx from the tip in the flow-through system at 38 °C. Curves 1–4 represent parallel measurements of one and the same experimental series.

Experiments with the 10^−4^ M BSA solution revealed no charge accumulation in the measuring cell at 38 °C (Figure 7). 

## 4. Discussion

Electrokinetic phenomena, for example, have been observed in water and water solution flows over polymer surfaces [14]. They are associated with the formation of a binary electric layer in an electric field and the movement of ions upon liquid phase drift over the stationary phase when a pressure gradient is applied. A similar movement of mobile ions is expected to occur under the influence of not only an electric field but also a pressure gradient. 

We observed an accumulation of positive charge in the measuring cell in water and 10^−15^ M protein solution flows inside a tubing with permanent pumping through the tip. In [11], a positive drop charge of ~0.1 nC was registered, and it was noted that a pipet tip was negatively charged.

Charge generation and accumulation data obtained upon charge measurement during the permanent influx of water and protein solution into the measuring cell are presented in Table 1.

Analysis of the obtained data implies the following: (1) the time dependence of a generated charge accumulation upon a permanent water influx into the measuring cell can have both a linear and an abrupt character; (2) similar sharp increases in charge accumulation time dependence are observed at both 35 °C and 38 °C, with similar values in the charge influx from the injector tip of about 3–8 nC/min. 

These sharp increases in the time dependence of charge accumulation in the measuring cell might be caused by either of the following: (1) the penetration of charged polymer particles from the AFM system delivery tubing walls and (2) changes in water structural characteristics arising in the water flow along the delivery system due to water–polymer surface interactions. Control AFM experiments performed earlier did not reveal an inflow of polymer particles with water or protein solution into the measuring cell [2,3]. The registration of not only a linear time dependence of charge accumulation but also sharp increases in charge value, both in water and femtomolar protein solution flows, suggest the existence of an electrohydrodynamic barrier to the charge influx into the measuring cell. The physical nature of this barrier may be linked with transitions between water ortho- and para-states. These transitions are highly possible in the phase transition zone at 35–37 °C and define a complex heterostructure of water and its anomalous properties, such as electroconductivity and viscosity in its outflow from the tip [13,15], which, in turn, causes a spike in charge outflow. Charge retention near the tip edge in the form of a charge cloud, as well as reversed conductivity (corresponding to the charge drain from the pipet tip outlet under the difference of potentials between the tip outlet edge and the grounding electrode Eg), may be associated with a complex water structure presented as a set of clusters. When this water structure is disturbed upon its outflow in an injector system, a sharp change of conductivity can appear. It should be noted that similar sharp changes in generated charge accumulation were observed in the 10^−15^ M protein solution. Water specific conductivity at 35 °C is about 0.1 × 10^−6^ S·cm^−1^ [16]. The specific conductivity of the 10^−15^ M BSA solution can be approximated from the specific conductivity value of the 10^−10^ M BSA solution, which is 50 × 10^−6^ S·cm^−1^ [17]. Conductivity of the 10^−15^ M BSA (λ_BSA_) solution, obtained via linear approximation of data taken from [15,16], has a value approximately 0.1% higher than that of the water specific conductivity value λ_H20_. This explains why the charge generation speed is similar both in water and in the 10^−15^ M BSA solution. The specific conductivity of the 10^−4^ M BSA solution is approximately 10^−4^ M/10^−15^ M = 10^11^ times higher than that of λ_H20_, so the «reverse current» corresponding to the charge drain from the pipet tip outlet under the difference of potentials between the tip outlet edge and the grounding electrode Eg substantially contributes to the charge change at the tip outlet, making it close to zero. This explains why no charge accumulation was observed in the measuring cell upon the influx of the 10^−4^ M BSA solution. 

Earlier we noted that low-copy (10^−15^–10^−17^ M) protein fishing in the AFM system is accompanied by charge generation [2,3]. Herewith, a generated charge increase in relation to the protein molecule number correlates with the enhancement of protein fishing efficiency by an AFM chip, which eventually influences the efficiency of protein detection. The revealed non-linear time dependence of generated charge accumulation in the liquid flow through the AFM protein fishing system should be taken into account in other biosensor systems, especially in nanowire detector-based biosensors, where the charge states of solvents and solutes influence detector conductivity. This effect can enhance protein fishing; on the other hand, it may result in incorrect protein concentration measurement. 

## 5. Conclusions

We showed that the time dependence of the charge outflow generated in the 10^−15^ M solution of water-soluble protein (BSA), permanently delivered to the AFM measuring cell from the flow-through injector, can have a non-linear character, with observed sharp increases. The emergence of such increases depended upon protein concentration. This effect was observed at both physiological temperatures of 35 °C and 38 °C, which are usually employed in highly sensitive analytical systems for biomolecule detections (AFM fishing of low-copied proteins, nanowire etc.). This effect needs to be taken into account while developing flow-through analytical systems for low-copy biomacromolecule registrations, creating enzyme kinetic models in hydrodynamic systems, developing a theory of hemodynamics with consideration of electrokinetic effects in flowing water and protein solutions. 

## Figures and Tables

**Table 1:** Dynamics of charge accumulation in the measuring cell upon permanent drop-wise influx of water or protein solution out of the AFM-fishing system injector tip.

Temperature	Type of Charge Accumulation	Water	10^−15^ M BSA Solution	10^−4^ M BSA Solution
T = 35 °C	Slow increase	0–0.3 nC/min	0.1 nC/min	0.1 nC/min no abrupt charge accumulation
	Sharp increase	1–4 nC/min	4 nC/min	
T = 38 °C	Slow increase	0–0.12 nC/min	0.2 nC/min	no charge accumulation
	Sharp increase	3–4 nC/min	3–8 nC/min	
